# ‘Stuck in catch-22’: a qualitative study of perceived work ability and decision-making about employment in severe asthma

**DOI:** 10.1186/s12890-025-03499-y

**Published:** 2025-02-17

**Authors:** P. Mackiewicz, H. Hussein, A. H. Mansur, M. T. Krishna, G. I. Walters

**Affiliations:** 1 School of Psychology, University of Birmingham, Edgbaston, B15 2TT UK; 2 Birmingham Regional NHS Severe Asthma Service, Birmingham, UK; 3 Institute of Immunology and Immunotherapy, University of Birmingham, Birmingham, UK; 4 Birmingham Regional NHS Occupational Lung Disease Service, University Hospitals Birmingham NHS Foundation Trust, Birmingham, UK; 5 Institute of Applied Health Research, University of Birmingham, Birmingham, UK

**Keywords:** Severe asthma (SA), Work ability, Employment

## Abstract

**Background:**

Severe asthma (SA) is characterised by persistent asthma symptomatology despite adherence to intensive treatment and control of asthma triggers. It is estimated that approximately 50% of individuals with SA have current employment, considerably less than rates in the general population. Poor physical and mental health status have been suggested as a potential explanation for this, but the relationship has not been investigated in depth. We investigated how bio-psycho-social and cultural factors influence work ability and employment in SA patients.

**Methods:**

Participants were recruited from the Birmingham Regional NHS Severe Asthma Service via opportunity sampling, with the exclusion criteria being individuals who had never been employed, currently in full-time education, or non-English speakers. Subsequently, men and those with minority-ethnic backgrounds were purposefully selected to gain a balanced sample. Interviews were performed either face-to-face, online or via telephone, transcribed using software and edited manually. Data were analysed using Reflexive Thematic Analysis.

**Results:**

The study included 12 participants (9 females and 3 males). Four major themes were constructed: impact of patients’ asthma control on work, psychological burden of living with SA, costs and benefits of being in employment, and adaptations and strategies for remaining in employment.

**Conclusions:**

Our findings highlight the potential for physical, occupational, psychological, and social support to address the diverse job-related difficulties experienced by people with SA. Additionally, national policy reforms should be considered to improve work capacity and promote employment opportunities.

**Supplementary Information:**

The online version contains supplementary material available at 10.1186/s12890-025-03499-y.

## Background

Severe asthma (SA) impacts approximately 5% of all adults with asthma and accounts for most of the United Kingdom National Health Service (NHS) asthma burden [1]. Individuals with SA experience persistent uncontrolled symptoms, frequent severe exacerbations that require systemic corticosteroids, asthma attacks and/or airflow limitations, despite high treatment demands and addressing contributory factors, distinguishing them from those with mild-moderate disease [2]. Deteriorating health status, and poor health-related quality of life are all recognised consequences of uncontrolled asthma and may lead to work disability. Moreover, occupational factors frequently aggravate asthma; these vary according to distribution and individual susceptibility.

Previous research on work ability and employment in asthma aimed to measure workers’ productivity, measured by absenteeism (time off sick), presenteeism (working at reduced capacity while unwell) and complete work cessation, grouped by variables such as asthma control and/or severity, often not investigating the issue holistically [3–5]. A previous study in our SA population revealed a current employment rate of 53%, lower than expected, and significant associations between unemployment and major comorbidities, anxiety and depression, female gender, social deprivation, and low asthma control and quality of life [6].

Furthermore, work-related exposures such as high physical demand and inhaled chemicals, are associated with increased productivity loss in SA [7–9]. In accordance with bio-psycho-social model of understanding disease or suffering [10] and later updates to include cultural elements [11], we aimed to understand how biological, psychological, social and cultural factors influence work ability and employment in SA, using qualitative methodology. Cultural norms such as beliefs and attitudes towards chronic health conditions could be particularly important for work outcomes in SA. To our knowledge this is the first such qualitative study.

## Methods

### Study design

We used a descriptive qualitative design with semi-structured interviews developed for this study (see Supplementary file). The interview schedule was developed by research team members PM, GW and HH, and was informed by combined clinical and research experience and published literature [3, 8, 12], to gain in-depth insight into SA patients’ experiences of work and employment. The final schedule was agreed by consensus between research team members. Reflexive Thematic Analysis was employed due to flexibility of the approach, relatively large and heterogeneous sample size to capture diversity, focus on providing a rich thematic description of our entire data set, and interest in how personal experiences are located within wider socio-cultural contexts [13].

### Participants

12 participants with SA were recruited from the Birmingham Regional NHS Severe Asthma Service (BRSAS) for qualitative interviews (see Table 1). We approached patients from diverse socio-demographic backgrounds, with various disease phenotypes and comorbidities and excluded those who: had never held paid employment, had not worked more than 10 years, were in fulltime education, or were unable to give informed consent or complete an interview in the English language. Participation was voluntary; however, we offered to reimburse any incurred travel costs.

**Table 1:** A summary of participant characteristics

Participant	Age	Ethnicity	Gender	Education	Employment status/type	Asthma phenotype	Comorbidity	Allergies	Smoking	Alcohol
P1	55	White British	Female	Secondary education	Unemployed/previously hospitality sector	Eosinophilic	Other non-asthma related comorbidity	Pollen, Latex, Milk	Former smoker	No
P2	40	White British	Female	Secondary education	Employed/finance	Eosinophilic	Other non-asthma related comorbidity	Pollen, Pets	No	No
P3	28	White British	Female	Higher education	Employed/finance	Eosinophilic and Infective	ILO, other non-asthma related comorbidity	Pollen, drugs (NSAIDs, aspirin, morphine)	No	Occasional
P4	51	British Pakistani	Female	Further education	Employed/social care	Infective	COPD Other non-asthma related comorbidity	Nil	No	No
P5	60	White British	Female	Secondary education	Unemployed/previously production and hospitality sectors	Eosinophilic	Other non-asthma related comorbidity	Pollen	No (passive smoking at work)	No
P6	64	Black British	Male	Further education	Employed/public sector and sport	Infective	Bronchiectasis, EDAC Other non-asthma related comorbidity	Pollen, dust, pets, dust, food allergies,	No (passive smoking at work)	Occasional
P7	52	White British	Female	Secondary education	Self-employed /animal care	Eosinophilic	No	Dust, pets, detergents	No	No
P8	66	White British	Female	Secondary education	Retired/hospitality sector	Eosinophilic	Other non-asthma related comorbidity	Pets, drugs (Aspirin, Penicillin, Methotrexate)	No (passive smoking at work)	No
P9	36	British Pakistani	Female	Higher education	Employed/administration	Eosinophilic	ILO, Rhinitis Other non-asthma related comorbidity	Pollen, pets, detergents	No	No
P10	61	Afro-Caribbean	Female	Higher education	Employed/education	Infective	Other non-asthma related comorbidity	Dust, food allergies, paint, pets, pollen	No	No
P11	35	White British	Male	Secondary education	Self-employed/constructions	Eosinophilic	ILO	Pets, dust, pollen	No	No
P12	77	White British	Male	Further education	Retired/law enforcement	Not determined	Other non-asthma related comorbidity	Dust	No	Daily

ILO = inducible laryngeal obstruction; COPD = chronic obstructive pulmonary disease; EDAC = excessive dynamic airway collapse; NSAID = non-steroidal anti-inflammatory drug

### Procedures

The study was sponsored by University of Birmingham and Health Research Authority granted ethical approval (REC reference = 22/PR/0096) on 17/02/2022. We employed opportunity sampling [14] via referral of eligible patients from the clinical team; the consent process and interviews were undertaken by the primary author. Informed consent to participate was obtained from all the participants in the study. Following interim recruitment review, the final 4 participants were purposefully selected based on their gender and ethnic identity. Recruitment and interviews took place between July and September 2022. A mutually convenient time and location were arranged to conduct interviews either face-to-face (*n* = 7), online (*n* = 1) or via telephone (*n* = 4). Interviews were audio-recorded using a digital Dictaphone (or Microsoft Teams), transcribed using Microsoft Teams and edited by hand by the primary author, to ensure accuracy and anonymisation. Interviews lasted between 29 and 93 min. Written field notes were made after the interviews to document observations and reflections which supported data interpretation. All electronic study data (audio files, coding, thematic analysis) was kept on a drive in a password-protected folder; no patient identifying details was kept with these data, only study participant numbers. Data processing was undertaken using NHS desktops. Completed consent forms were kept in a separate site file and destroyed within 3 months of finishing the study. No participants withdrew from the study.

### Data analysis

**Fig. 1:**
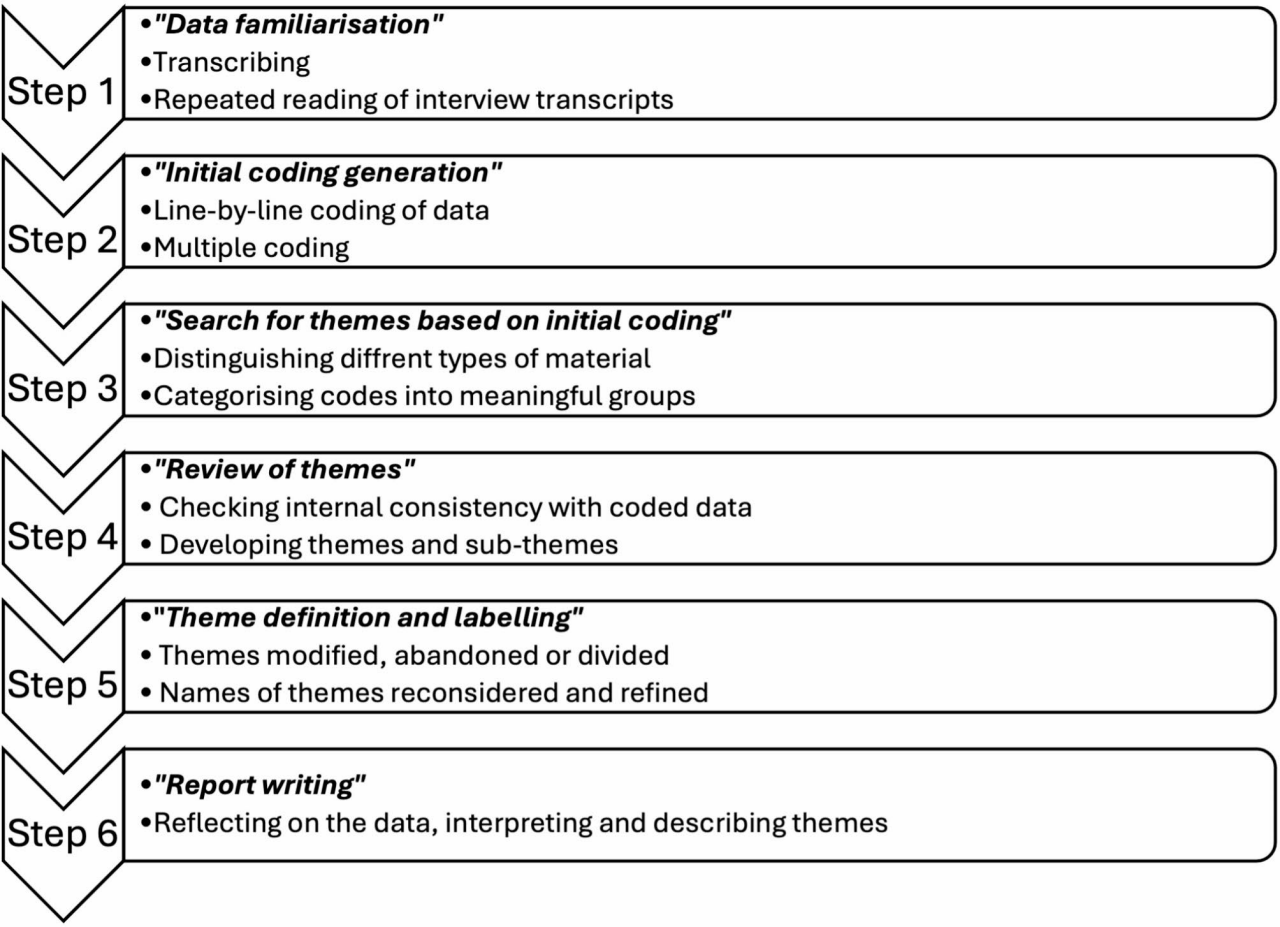
Description of the stepwise reflexive thematic analysis process adopted; after Braun & Clarke [13]

**Fig. 2:**
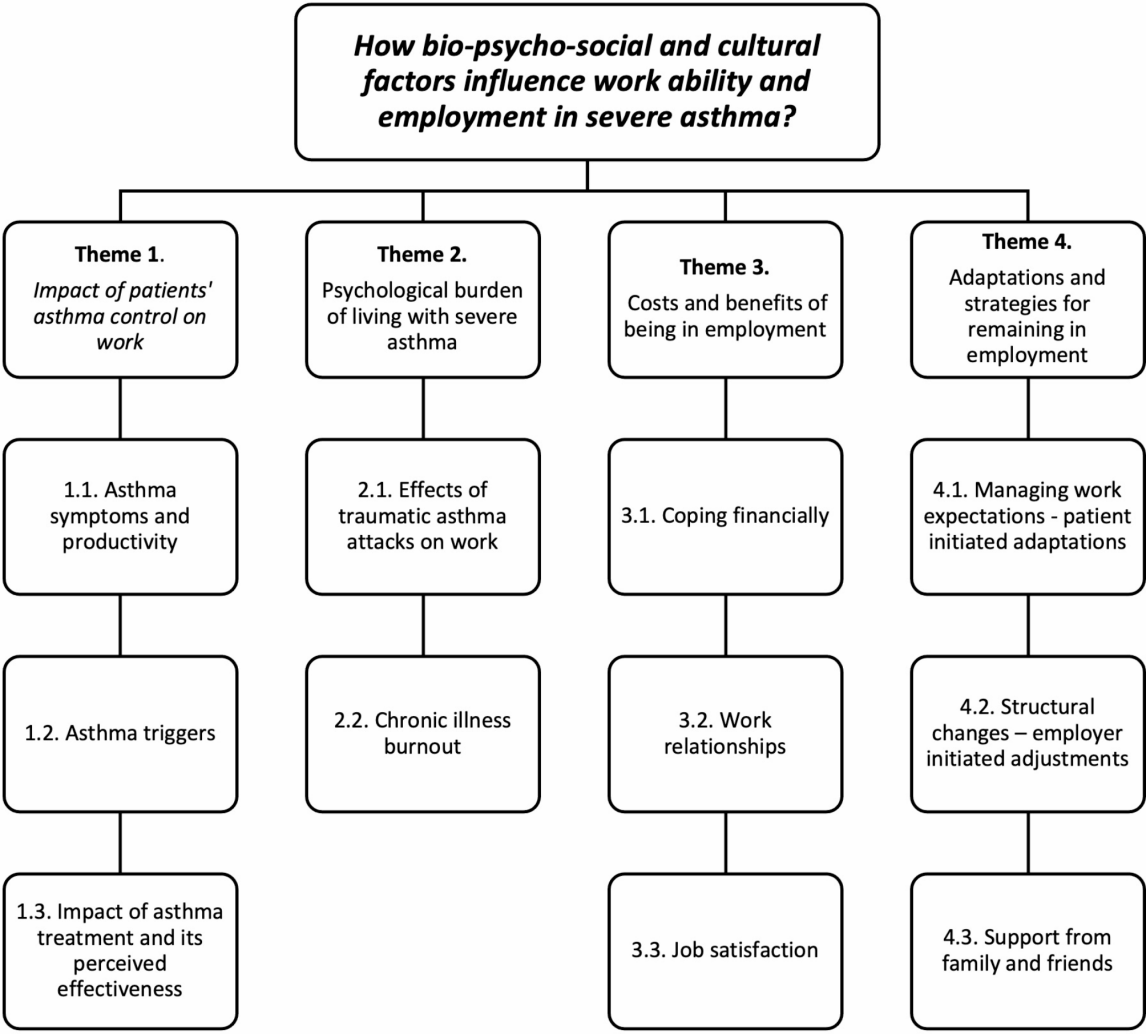
High-level themes and subthemes identified through thematic analysis

We chose the critical realist and epistemic contextualism position for analysis; this is a critical position towards objective reality, with individual perceptions determining what is accepted as truth [15] and knowledge gained via broader socio-political and cultural context, that impinge on how patients make meanings of their experiences. A systematic approach to thematic analysis was followed [13, 16, 17], (Fig. 1). Given the broad research question and limited previous research on this topic, inductive coding of the entire data set was undertaken; however, some coding was also informed by published quantitative research and supervisors’ clinical expertise. Multiple coding [18] was used to cross-check coding strategies and interpretation of the data across three interviews, and concordance discussed. Investigator and theory triangulation was used to interpret results of the study. Individual thematic maps were discussed, refined in relation to the research question, and final high-level themes and subthemes created (see Fig. 2). No minimum frequency was set, but themes were constructed from codes that were prevalent across multiple participants.

### Reflexivity

The primary author (PM) was a postgraduate student researcher with no prior qualitative research experience. She had no established professional or personal relationship with any participant. The researcher’s held belief that most people can find and sustain employment providing appropriate adaptations and training are given, may have led to a confirmation bias. Whilst all efforts were taken to ensure the interview questions were open and not assumptive, prompts were used to gather data deemed useful to answer the research question. Academic supervision was provided by (1) the senior author (GW) is a Consultant in Occupational Respiratory Medicine and Senior Research Fellow in Birmingham who has undertaken 2 previous qualitative studies exploring barriers and facilitators of identifying work-related asthma, and (2) co-author (HH), who is a Clinical Psychologist within BRSAS and has undertaken previous qualitative study of illness perception in patients with difficult-to-treat asthma. Regular supervision meetings were used to challenge biases throughout the research project and understand data from different perspectives.

## Results

Four high-level themes were generated (see Fig. 2); data codes are presented in the Supplementary file. Each theme relates to the bio-psycho-social and cultural factors that impact on work ability as well as the mechanisms that underlie them.

### Theme 1: impact of patients’ asthma control on work

This theme was constructed based on patients’ experiences of poorly- versus well-managed asthma symptoms at work (also see Table 2). Participantss with uncontrolled asthma described it as a vicious cycle of being poorly, seeking medical attention, feeling better, engaging in physical activities, and becoming unwell again. Physical activities, low energy levels, work stress and environmental exposures appeared to be major barriers to continuing employment. Properly selected medication and adherence to a healthy routine had the potential to enhance work capacity. However, side effects of medication and seasonal asthma worsening were also common and affected work ability.

**Table 2:** Illustrative quotes for theme 1: impact of patients’ asthma control on work

I have a lot of time off work and I find that due to like financial situations and stuff I do have to sometimes put my asthma and my symptoms on the back burner and I could go to work, because there’s nothing like you got… but I also got to think about my health and like my medical history of what’s happened, with like the comas and stuff like that. So it is very hard and it even at the meetings they ask me ‘do you feel like sometimes you don’t give your body enough rest to get over stuff’ and I was like yeah well because I can’t afford to be off for this long because of how often it happens. (P3)
Well because asthma interrupted my sleep pattern and I wasn’t able to work to the same efficiency, having spent a night mostly without sleep. And it affects your demeanour, as well, and your concentration…You can’t really put a comprehensive report together if you’re feeling tired and lethargic. So, you’d have to put it to one side and do it again. (P12)
I’ve taken a lot of sick time…I’d want to have a full-time job, I couldn’t do that without actually falling behind on work and then going back and being stressed about it, which would in turn trigger me again… or like if you’re doing physical exercise, things like having a full-time job where you’re full time looking after your kids… and then also making sure I’m managing my asthma when I’m being able to breathe properly and having that time to be able to rest when I’m ill so I can get back to normal and do my normal activities. I don’t think I could do that with a full-time job (P9).

#### Asthma symptoms and productivity

It was evident throughout participants’ narratives that struggles with asthma symptoms made it difficult to stay productive at work, resulting in periods of presenteeism; “on a good day, I can do a lot more for example plastering, whereas if my asthma is bad it can take two or more days” (P11). The number of sickness absences per participant due to asthma was wide-ranging; for example, “prior to my last hospital admission in April, I haven’t been off at all. I’ve been there for two and a half years” (P2); others were absent for as long as six months in the previous year. Although participants recognised that looking after their health was important, they also reported that taking time off work to recover was not always possible, as they may struggle financially and fear losing their job; “sometimes you feel like you know, even if you’re not well, you have to make yourself well because you don’t want to lose your job” (P9). Phased return-to-work was not always adopted by the employer which led to further presenteeism and symptom worsening; “If I had a major asthma attack, within a few days they’d expect me to be back…nobody understood how you’re physically battled” (P7).

Conversely, patients with well-managed asthma symptoms and a good routine reported very good ability to do their jobs, but at same time acknowledged that; “there were times when, like after an asthma attack, all the exacerbations, when it was really difficult” (P6). The fluctuating and sometimes unpredictable nature of symptoms changed patients’ ability to work; “unless I’ve got a cold, and my asthma is playing up… Other than that, I’m fine working” (P10).

#### Asthma triggers

Almost all participants were able to identify their asthma triggers. These varied between individuals, according to the nature work, and whether or not they were perceived to be out of their control; “if I get really hot or damp, see dust it’s weird with dust or animals, you put a mask on and I get hot so either way I struggle anyway… you’re sort of stuck” (P11). Patients recognized many environmental triggers such as smoke, pollution, perfumes, cleaning products, strong smells (e.g. glue), dust, damp, feeling hot or cold, infections and physically demanding tasks. Worrying about control of external factors caused stress and anxiety, therefore that process itself was identified by most patients as an asthma trigger. Work-related absences and falling behind work were identified as major threats and often created a vicious cycle of sickness, falling behind, absence meetings and stress, triggering sickness absence again. Moreover, sometimes people felt that maintaining full-time work created too much pressure to be able to manage SA, especially those with domestic and caring responsibilities.

#### Impact of asthma treatment and its perceived effectiveness

From the early stages of diagnosis, participants used strategies to help them cope with their SA symptoms. Patients’ narratives conveyed a sense of confidence in treatment efficacy, as well as challenges met on their journey to finding the right medication regime and dealing with side effects. It was evident that not only asthma symptoms themelves but also steroid treatment had detrimental effects on patients‘ levels of productivity; **“** “My journey to actually find the medication that works and I’m not allergic to was a very bumpy one…and when I’m on the steroids, that makes me immensely tired, really tired. That’s why I avoid those like the plague.” (P10).

Whilst patients put a lot of trust in the multidisciplinary SA team, they also acknowledged the importance of personal investment, including good routine, looking after themselves and adherence to medication in order to keep their asthma under control; “every piece adds to staying in control, so I make sure I get seven hours sleep and make sure I’m not overtired, and I do keep on top of my weight because it did affect my asthma.” (P7). Nevertheless, sticking to a routine was not always possible in roles that are unpredictable, such as a police officer. Having medication available at work was another factor contributing to staying in control of asthma. Nevertheless, feeling judged and shamed were some of the barriers to taking medication in public places; “Because you feel like you’re being judged. I might not be, but in your head it’s that sort of way because you meant to be strong.” (P11). Being aware of the brittle clinical features of asthma, getting help and administering medication on time, as well as not downplaying symptoms by both staff and patients, seemed vital in avoiding serious asthma exacerbations.

Biologic injections and inhalers were also perceived as effective in managing asthma and improving physical fitness. Nevertheless, it was noticed that their effects can also wear off; “he tweaked my inhalers and that helped for a little bit. But then I don’t know, it just started to go a bit haywire.” (P8). Moreover, biological treatment required taking time off work fortnightly for administration, which for some meant reduced pay or arranging cover for their absence.

### Theme 2: psychological burden of living with severe asthma

Patients’ struggles with finding work-life balance, good health and ‘normality’ threatened their psychological wellbeing. This was evident in narratives about fear of asthma and panic attacks; “and if I was out and I hadn’t got an inhaler in my handbag, I wouldn’t enjoy the night. I’d be panicking that I was going to go into an asthma attack. It’s horrible” (P7). Other psychological difficulties were evident: post-traumatic stress, feelings of isolation, shame, and low mood and self-esteem; “…put everything into one bag, it just makes me depressed.” (P4). Participantss also found themselves feeling frustrated about asthma limitations “it was getting on my nerves more than anything” (P8), leading to chronic illness burnout. Also see Table 3.

**Table 3:** Illustrative quotes for theme 2: psychological burden of living with severe asthma

My issues with the two induced comas, umm I was actually seeing a psychologist for PTSD from it and at work it was a major struggle because the sound at the checkout… will immediately take me back being in recess like with the noise of the machines… all the stress when I was doing, being in ICU and stuff, so that was really, really tough for a while. (P3)
Everybody thinks we can’t breathe and that’s it. They don’t realize how it dominates your life. And mentally dominates your life because I became frightened to do things. I was frightened to go back. If I’d forgotten inhaler, I’d have to come home. (P7)
I was so poorly and at home, and I couldn’t do anything and go anywhere, I was just kind of stuck because I wasn’t well enough to go and then when I did feel a little bit better, I was too scared to go out anyway, in case I had an asthma attack because I’ve been in attacks, I had asthma attacks when I’ve been out… I was worried all the time about my breathing. I was just like, there’s so many people there, it’s embarrassing being taken off all the time in an ambulance. (P1)
You can’t do it. Yeah but I’ve always done it. Yeah, but you can’t do it now, can you? And then I’ll get angry at them then, and I’ll be saying shut up and don’t tell me what to do, I can do it. But to be told you can’t by somebody until you’ve accepted it yourself you can still get angry, yeah… It eats you away cause you’re thinking I’m feeling useless. So, I stopped, finally (crying)… I used to, like, cut myself off and switch off, you do get more selfish to allow time for yourself… (P8)

#### Effects of traumatic asthma attacks on work

Life-threatening asthma attacks appeared to have persisting effects on patients’ day-to-day functioning; “I won’t go because fear come to ya [sic], sometimes thinking just in case something happens to me outside, are you really fine?” (P4). Participantss recognised that looking after their mental health was important for managing their asthma; “I have to make sure I see positives and I have good mental health, not just physically but mentally as well. So, I’m not putting that pressure on myself and causing myself inflammation and stress.” (P9). Moreover, having asthma attacks in public places like work felt ‘embarrassing’ therefore, patients became preoccupied with avoiding distressing situations, which consequently resulted in partial or complete work cessation.

#### Chronic illness burnout

Living with SA required consistent and daily effort to manage the condition. Participantss reported feeling; “tired of being ill or going to hospital appointments all the time…” (P11). Moreover, being restricted in activities brought up feelings of anger, low mood and affected their self-worth; “the mental side for me is worse than the physical. I can struggle all day long, but it’s mentally, it drains you” (P11). It was evident that patients wanted to maintain ‘normality’ and ‘independence’ by continuing to work; “Yeah I just want to live my normal life, you know I don’t wanna be a burden on anyone, not even my own partner. I want my own independence… Want to have my own money, not to ask anyone.**”** (P4), and when the burden of SA affected their ability to do so, it led to deterioration in mental health, which affected patients’ confidence to work.

Although, mental health distress seemed common among SA patients, stigma around getting psychological help was a barrier to receive support; “I was put forward to see a psychologist, in the beginning I didn’t want to know. I said no, I’m not mad…but then, I thought I do need to talk to someone. Best thing I’ve ever done.” (P8). It became apparent that working with SA was challenging, required a lot of decision making, and prompted people to weigh the pros and cons of continuing to work.

### Theme 3: costs and benefits of being in employment

Participantss’ stories illustrated the search for balance between looking after their health and maintaining ‘normality’; “I’m still trying to carry on, do as much as I can and sometimes, I do push myself, so it’s trying to find that balance and accepting where my asthma is at and not pushing myself” (P3). The societal pressure to work, poor sick pay, difficulties accessing welfare support and participants’ own attitudes towards benefits could limit the possibilities of reduced work. This work dilemma was further complicated by company policies, management, colleagues and family understanding, and support provided to employees affected by SA. Also see Table 4.

**Table 4:** Illustrative quote for theme 3: costs and benefits of being in employment

I tried for PIP [sic; personal independence payment] a few months back and then I think I scored four….Yeah I wanted support but I didn’t get it. I can’t. I want to but you know, with kids and stuff like my house, I have to do what I do… So, like I said, I tried for PIP so if I am ill, I can have a day off, but I’m not entitled to a lot when you’re self-employed. So it’s an awkward one because it’s not like, because I still go to work, it probably knackers my chances of getting any help… (P11)
My boss is just the best. She is incredible. And she’s been so understanding about everything the last few months. And she she was more than happy for me to work from home, but she tells me to take it easy. Yeah, I’m incredibly lucky, which obviously helps…I’ve always felt very comfortable in the office, and they know, they know the situation surrounding my asthma. (P2)
It took a few years before it started to sink in, you know, and then it was when I actually said the words to myself, you’re killing yourself, you’re literally killing yourself. But when you work from the time you leave school and always been busy and always done stuff. The hardest part is knowing that you can’t do it, up here in your head you think you can, but you can’t, your body tells you can’t… You lose your identity. A little bit. You lose who you are… (P8)

#### Coping financially

Participantss identified that reducing their hours, having adequate sick pay and disability benefits would be helpful in attaining rest and looking after their health better. Nevertheless, financial pressures and personal circumstances often did not allow them to follow that path. Unsuccessful benefits claims and feeling pressured to work despite ill health, made patients feel alone in their struggles. Indeed, participants that worked reduced hours felt “…lucky because at least I can afford to work part-time and look after my health better” (P10). Conversely, some SA patients did not consider themselves disabled and would not accept financial support. Patients’ attitudes towards benefits and values impacted SA management as participants could be “…putting a lot of pressure on myself to try and work as hard as normal person would…” (P8).

#### Work relationships

Where employers were perceived as understanding of SA and its impact on the person, it was interpreted as practically helpful (e.g. in allowing more time to recover) and empowering for participants. For those self-employed, having an escalation plan and ensuring stakeholder (e.g. customers) awareness of SA was seen as important in order to get through flare-ups.

Nonetheless, a majority of participants reported that their SA was often poorly understood and they felt ‘judged’ and disbelieved by co-workers and managers; “You know if you go to work and you’re not feeling well, they’ll be looking at you or you know, you’re not very productive.” (P4). Strikingly, some participants explained that having an asthma attack at work was somewhat eye opening for employers. Lack of employer consideration surrounding SA was a reason for some participants to leave a job; “…because to say I can’t work with this cleaning product and stuff like that, they got very uppity. They were quite ignorant towards it, so I quit.” (P7). The lack of understanding and support was especially evident when managers did not consider the occupational health advice, made employees feel threatened upon return to work through absence warning processes, and discriminated against internal promotions. Discrimination in the workplace was a big concern for people with SA, with many participants perceiving they had lower chances to find another job as; “…they don’t stand a chance over someone healthy.” (P11). Moreover, with intersectionality coming into play, the barriers to employment were compounded by other protected characteristics.

#### Job satisfaction

Most participants were committed to work and expressed a strong desire to continue working and live a life aligned with their values. Work provided meaning and purpose in life for many participants and was or still is a big part of their identity; “Well, I went to work because I enjoyed it. I found I found it challenging. And, you know, very satisfying.” (P12). Participantss feared losing their work identity and found it very hard when they had to leave or change jobs due to health; “I couldn’t have maintained it with my health…It’s upsetting because they were things I kind of, like created, my babies. That was very painful because that was something I didn’t want to give up, but I did have to.” (P9).

Feeling valued at work contributed to staying in employment, kept people motivated and was beneficial; “…in social terms, in terms of your self-worth you know” (P10). Unfortunately, many participants felt undervalued, their job was unrewarding and generally they felt dissatisfied with their employment; “I go to work, to pay bills with some satisfaction in it, but not much these days…” (P10).

### Theme 4: adaptations and strategies for remaining in employment

Despite numerous difficulties SA patients faced at work, they demonstrated incredible strength and resilience in their professional lives and continued to learn methods to stay and cope in employment. Employers, as well as patients’ networks of family and friends played an important part in adjustments and support to remain in work. Also see Table 5.

**Table 5:** Illustrative quotes for theme 4: adaptations and strategies for remaining in employment

It is the first time in many years I’m feeling more confident in myself. Seeking out to get like a Camper Van type of like take away things for my baking, it would be something around my family business and it’s something I’ll enjoy more… 6 h is my goal and I can do it for another day or two and just taking the time out making sure in between everything else in the house is up to date, nobody is like missing out or I don’t feel stressed about anything and I still can maintain everything else. So, if I’m not well one week… I can still go back and just carry on with it after a couple of weeks… (P9)
Committee members that used to say to me when I had a few episodes, why don’t you let us come and help with the caller… And as I said, the girls, both my girls were trained as well…I was lucky with that side of the help as well. I think that helped me stay as long. I am lucky compared to some that really suffer. Just got them to fall back on there… (P8)

#### Managing work expectations - patient initiated adaptations

Majority of participants recognised that living with SA often means learning how to work with their condition and not against it. Accepting limitations allowed patients to manage expectations and adapt accordingly; “I can’t look after myself if I had a full-time job… I think why I even became in this position is because I didn’t put my health first” (P9). Nevertheless, recognising and accepting asthma limitations was not straightforward; “the biggest, the hardest part is saying to yourself, I can’t… once I started getting on that track, then that did help a lot more” (P8). Participantss often tried to carry on as normal and expected more of themselves. This experience was especially apparent among men; “Thirty-three-year-old, six-foot-three bloke should be able to do most things and I’m restricted. I don’t like that… generally men don’t want to admit they’re knackered, and I don’t like letting the bloke I work with down. Men are different” (P11). Unsuccessful attempts to carry on as normal can often resulted in negative feelings; “…this is getting on my nerves…and then I’d have to sort of lean against the wall or maybe pretend I’m looking for something. I’m feeling bad cause I’m thinking you have to just stop.” (P8).

It was evident from participants’ narratives that accepting asthma limitations helped to live a valued life and regain confidence, despite changes in their usual way of doing things; “I’m building up capacity in my head and physically slowly, so I can put it into something that I can do full-time and it’s for me and I enjoy. I can’t do it straight away and I know that.” (P9).

#### Structural changes– employer-initiated adjustments

Participantss recognised that reasonable adjustments under the Equality Act [19] allowed them to do their job despite their disability constraints. Patients identified many adaptations that helped them stay in employment, namely changing hours, changing routine, having extra time for completing tasks, taking extra breaks and working flexible hours. Participantss also could work from home whenever possible and some people had a higher absence allowance; “Normally they give absence percentage as 2, I think it’s 2%, but through occupational health they pushed mine on up to 5%.” (P3). Employers also tried to limit infections during COVID-19 with various workplace measures (e.g. masks, remote working, limited social mixing) and introduced accessibility adjustments in workplaces such as parking and lifts. Moreover, some employers changed patients’ roles to be more responsive to their SA; “I first started uhm, working as a part of the grocery team checking cells, then I moved to stock control due to my asthma and my health and they moved, I moved up to wages.” (P3). However, some participants perceived that not all employers adhere to the Equality Act [19] as they were never referred to occupation health and were asked to retire early instead; “Nothing was put in place ever to help me, perhaps at the very end something could have been. they only brought me in to speak to me about having to retire early because of my health.” (P6).

Self-employment was also seen as a more flexible form of employment as participants could make most decisions and accommodate time for treatment; “If I was employed then would they accept your time off or not all the time, probably not for appointments… you have flexibility when you’re self-employed.” (P11). However, self-employment could be difficult when it came to taking time off sick; “Sometimes I shouldn’t go to work, but when you’re self-employed you still have to because I don’t get help.” (P11).

Despite numerous adjustments that a majority of participants had in place to continue working, some recognised that certain job roles could not be adapted to suit their needs; “It’s not a sort of job that you can just say I can, I can just go in there, do 4 hours and go home, it’s not that sort of job.” (P8), “There’s no way of avoiding it (triggers), you understand. I do enjoy my work I’ve been looking at changing jobs because of the way it affects me, but I like what I do, I’m a practical person.” (P11). Educational background was also a barrier for people wanting to change jobs; “I don’t think I could even hold down a job now if I’m honest because…I huff and puff all the time and I don’t know how to do computers and things like that.” (P5).

#### Support from family and friends

Several participants identified having a network of people offering support - practical help with work tasks; “I always get a friend to come and sit with them (dogs) while I’m out getting the injections” (P7), financial help when off sick, and emotional help to deal with asthma challenges; “He’d breathe with me, yeah, which was always really good. It would bring you down. He was there like, I’ve got amazing children.” (P7). It appeared that help from family and friends also played an important role incontinuing to work with SA, highlighting that work outcomes were created collectively.

## Discussion

Our findings indicate that the impact of SA on work is immense, with absenteeism and/or presenteeism, early retirement, and job or role change all appearing regularly throughout the dataset. Nevertheless, the study also emphasises patients’ ability to adapt to health circumstances and live a valued life, in which employment plays a significant role. Structural elements, comprising occupational health provision, workplace health and safety measures, equity in employment opportunities, sickness and welfare policies, may aid SA patients in finding and sustaining employment. Although, the bio-psycho-social approach [10] tries to work towards a more holistic understanding of what may contribute to patient’s difficulties from a range of perspectives, it appears that experiences of work ability in SA extend beyond this model to include wider systems such as economy, austerity, cultural norms, healthcare resources and time [20].

Our findings support the existing literature on the detrimental impact of poor asthma control, workplace exposures, physical demands, severe symptom burden, corticosteroids use, depression and/or anxiety on ability to work [3, 4, 5, 6, 8–9, 12]. Participants in this study described the perceived benefits of biological treatments in alleviating the impact of the aforementioned factors. Other studies have also demonstrated the beneficial effects of biological agents in mitigating occupational impairment [21–23], which may also benefit the broader economy. This study also offers novel understanding of factors affecting work ability and decision making about employment, for example highlighting medication administration and challenges with maintaining healthy routines as barriers.

Psychological distress such as depression, anxiety, panic attacks and post-traumatic stress disorder are common in chronic diseases [24] and are associated with significantly higher levels of work impairment in SA [12]. We found that chronic illness burnout (here described as frustration with asthma limitations, fatigue, lack of confidence and motivation) negatively influenced work ability, a finding observed by others, and exacerbated during the Covid-19 pandemic [25]. It was evident from the data that people’s workplaces introduced various Covid-19 control measures such as limiting social mixing, wearing masks, furlough and remote working, which might have changed people’s perceptions of their ability to work. Furthermore, although infection risks have been controlled more effectively, this may have suited some more than others, and for some there was a risk to mental wellbeing through health anxiety, social isolation, financial problems and greater childcare demands [26]. Addressing psycho-morbidity leads to reduced corticosteroid use, better disease control and asthma-related quality of life [27]. Furthermore, on-site psychology support within SA services can reduce accessibility and stigma barriers [28].

Participantss weighed advantages and disadvantages of working with SA. Most felt pressured to work to meet financial obligations, and perceived that inadequate statutory sick pay and a difficult-to-navigate welfare system often forced them to work whilst unwell. UK statutory sick pay is one of the lowest in Europe, worth18% of average weekly gross pay [29–30], in contrast to other Norther European countries, many of whom pay between 70 and 100% of weekly wage.

This and other studies report instances of workplace discrimination faced by individuals with SA. These discriminatory practises often result in barriers to entry or promotion within certain professions, all of which may pose significant risks to affected individuals’ self-esteem and financial stability [31–33]. Even when SA symptoms improve, such negative work experiences may result in a cycle of continued work impairment.

Our findings closely resemble the work ability model conceptualised as a balance between one’s resources (personal, structural and relational), work demands, and the impact of health impairment and motivational process [34]. Our analysis indicated the importance of good health and wellbeing, realistic professional goals, receiving support from social networks, as well as workplace management and colleagues’ understanding to enable introduction of work adaptations; all of the aforementioned feed into job attitudes and performance. Our study suggests that individuals who perceive a high level of support in their workplace tend to show more commitment, improved performance, and feel satisfied and valued. Moreover, the support and understanding received from others can influence one’s own acceptance of living with a chronic condition, reduce the feelings of guilt, shame and anger, and instead enhance individual’s self-confidence and prevent from losing their professional identity, which serves as a source of meaning and purpose for many.

Work-related exposures as causes or triggers for asthma are often overlooked by primary and secondary healthcare professionals [35]. Occupational asthma accounts for 1 in 6 cases of adult-onset asthma, a significant proportion of which is severe or difficult-to-treat and ongoing exposure to the cause can result in accelerated lung function loss and poor employment outcomes [36]. Work-exacerbated asthma is a prevalent concern impacting < 25% of those with pre-existing asthma [37]. Early enquiry and elimination of workplace exposures are therefore crucial for effective treatment. Moreover, routine collection of data on job role and work-relatedness in the national SA registry, would yield significant knowledge about the burden of work-related asthma [38].

### Limitations

Our sample is broadly representative of the UK SA population, where 63% of entries in the national registry are women [39]. 30% of our cohort included patients from Ethnic Minority Groups, where clinical outcomes of asthma have been reported to be poor [40]. However, our cohort did not include those who had limited or no proficiency in speaking in English, and this is a potential limitation to our findings. We have not accounted for gender differences in workplace exposures; that is, men may have experienced both employment and work ability differently due to (i) more physically demanding and dangerous work exposures in industries such as constructions and manufacturing, where there is a male predominance, as well as (ii) the ingrained gender roles for men to be providers within a family, with some ethnic variations reported [41]. In addition, it should be noted that the job experiences of all participants were from the UK, limiting the transferability of findings to countries with different cultural and socio-economic contexts. Another limitation is that we have not accounted for major comorbidities, all of which have the potential to affect work ability. Moreover, the use of a single site for recruitment also can be considered a limitation. A possible selection bias due to participants being referred to the study by the clinical team could have occurred. We did not track data saturation to signal the end of data collection. The interview data was collected in person (*n* = 7), via phone (*n* = 4) and online (*n* = 1), nonetheless we have not attempted to compare whether the method of interview impacted the quality of data. Moreover, the interview guide was not piloted with patients prior to commencing the study.

### Recommendations

It is beneficial for patients to be aware of their legal rights in the workplace and speak with their employer to make sure they understand how SA affects their daily life. Making reasonable adjustments will benefit both employees and employers, as SA patients will be able to continue doing their job well with the right support in place. Patients may also be eligible for support through Access to Work grants and other schemes. Additional resources and financial support for managing SA at work can be found in the reference Sects [42–43]. It is recommended that future research includes employers to obtain an alternative perspective on the issue– how they view SA and employee’s ability to work, and challenges for businesses. SA patients would also benefit from future studies that develop, implement and evaluate interventions that support work involvement.

## Conclusions

Despite the small sample size, the purpose of this study was to explore the perspectives of individuals with severe asthma on how biopsychosocial and cultural factors affect work ability and employment. The impact of work impairment extends beyond the person and has far-reaching effects on employers, co-workers and the society, contributing significantly to the overall burden of asthma [4, 44]. Our study highlights the potential for physical, occupational, psychological, and social support, including policy reforms to address the wide-ranging work challenges patients face and enhance work ability. Additionally, there is a need for greater public awareness and education about SA to minimize patient distress in the work settings.

## Electronic supplementary material

Below is the link to the electronic supplementary material.

Supplementary Material 1

## Data Availability

The datasets analysed during the current study are available from the corresponding author on reasonable request; these will be redacted to remove the possibility of identifying individual participants or employers.

## Abbreviations

SA: Severe asthma
BRSAS: Birmingham Regional NHS Severe Asthma Service
OCC: Occupational health
PIP: Personal Independence Payment

## References

[1] NHS England. Specialised Respiratory Services (adult)– Severe Asthma, Redditch UK. 2017. https://www.england.nhs.uk/publication/specialised-respiratory-services-adult-severe-asthma/. Accessed 13 Jan 2023.

[2] Chung KF, Wenzel SE, Brozek JL, Bush A, Castro M, Sterk PJ et al. International ERS/ATS guidelines on definition, evaluation and treatment of severe asthma. European Respiratory Journal. 2013;43(2):343–73. Available from: https://erj.ersjournals.com/content/erj/43/2/343.full.pdf 10.1183/09031936.00202013 24337046

[3] Hiles SA, Harvey ES, McDonald VM, Peters M, Bardin P, Reynolds PN, et al. Working while unwell: workplace impairment in people with severe asthma. Clin Experimental Allergy. 2018;48(6):650–62. 10.1111/cea.13153 29676834

[4] Sadatsafavi M, Rousseau R, Chen W, Zhang W, Lynd L, FitzGerald JM. The preventable Burden of Productivity loss due to suboptimal Asthma Control. Chest. 2014;145(4):787–93. 24337140 10.1378/chest.13-1619

[5] Vietri J, Burslem K, Su J. Poor Asthma control among US workers: health-related quality of life, work impairment, and health care use. J Occup Environ Med. 2014;56(4):425– 30. 10.1097/JOM.0000000000000123. PMID: 24662951. 10.1097/JOM.0000000000000123 24662951

[6] Walters GI, Marsh J, Bahron A, Hussein H, Krishna MT, Mansur AH. Associations between employment and sociodemographic and health-related factors in asthmatic patients assessed at a regional severe asthma service. J Allergy Clin Immunol Pract. 2022;10(6):1646–1648. doi: 10.1016/j.jaip.2022.02.031. Epub 2022 Mar 5. PMID: 35259536. 10.1016/j.jaip.2022.02.031 35259536

[7] Wong A, Tavakoli H, Sadatsafavi M, Carlsten C, FitzGerald JM. Asthma control and productivity loss in those with work-related asthma: a population-based study. J Asthma. 2017;54(5):537–42. 27494107 10.1080/02770903.2016.1220011

[8] Eisner MD, Yelin EH, Katz PP, Lactao G, Iribarren C, Blanc PD. Risk factors for work disability in severe adult asthma. Am J Med. 2006;119(10):884– 91. 10.1016/j.amjmed.2006.01.016. PMID: 17000221. 10.1016/j.amjmed.2006.01.016 17000221

[9] Blanc PD, Cisternas M, Smith S, Yelin EH. Asthma, employment status, and disability among adults treated by pulmonary and allergy specialists. Chest. 1996;109(3):688–96. Erratum 2000. 8617077 10.1378/chest.109.3.688

[10] Engel GL. The need for a New Medical model: a challenge for Biomedicine. Science. 1977;196(4286):129–36. 847460 10.1126/science.847460

[11] Hilty DM, Advancing Science, Clinical Care and Education. Shall we Update Engel’s Biopsychosocial Model to a Bio-psycho-socio-cultural Model? Psychology and Cognitive sciences -. Open J. 2015;1(1):e1–5.

[12] Ong ASE, Chan AKW, Sultana R, Koh MS. Impact of psychological impairment on quality of life and work impairment in severe asthma. J Asthma. 2021;58(11):1544–53. Epub 2020 Aug 24. PMID: 32777181. 32777181 10.1080/02770903.2020.1808989

[13] Braun V, Clarke V. Using thematic analysis in psychology. Qual Res Psychol. 2006;3(2):77–101. 10.1191/1478088706qp063oa

[14] Coolican H. Research methods and statistics in psychology, Sixth Edition. Florence: Taylor And Francis; 2014.

[15] Vincent S, O’Mahoney J. Critical realism and qualitative research: an introductory overview. The SAGE handbook of qualitative business and Management Research methods: history and traditions. London: SAGE Publications Ltd.; 2018. pp. 201–16. 10.4135/9781526430212

[16] Braun V, Clarke V. Successful qualitative research: a practical guide for beginners. London: Sage; 2013.

[17] Braun V, Clarke V. Reflecting on Reflexive Thematic Analysis. Qualitative Research in Sport, Exercise and Health. 2019;11(4):589–97. Available from: 10.1080/2159676X.2019.1628806

[18] Barbour RS. Checklists for improving rigour in qualitative research: a case of the tail wagging the dog? BMJ. 2001;322(7294):1115–7. 11337448 10.1136/bmj.322.7294.1115 PMC1120242

[19] Equality Act. Equality Act 2010. Legislation.gov.uk. 2010. Available from: https://www.legislation.gov.uk/ukpga/2010/15. Accessed 8 May 2024.

[20] Bronfenbrenner U. The ecology of human development. Cambridge: Harvard University Press; 1979.

[21] Javier Gonzalez Barcala F, La Fuente-Cid RD, Alvarez-Gil R, Tafalla M, Nuevo J, Caamaño-Isorna F. Factors Associated with a higher prevalence of work disability among asthmatic patients. J Asthma. 2010;48(2):194–9. 21142707 10.3109/02770903.2010.539294

[22] Zazzali JL, Raimundo K, Trzaskoma B, Rosén K, Schatz M. Changes in asthma control, work productivity, and impairment with omalizumab: 5-year EXCELS study results. Allergy Asthma Proc. 2015;36(4):283–92. 26108086 10.2500/aap.2015.36.3849

[23] Mansur AH, Srivastava S, Mitchell V, Sullivan J, Kasujee I. Longterm clinical outcomes of omalizumab therapy in severe allergic asthma: study of efficacy and safety. Respir Med. 2017;124:36–43. 28284319 10.1016/j.rmed.2017.01.008

[24] Asthma UK. What’s the link between lung conditions and mental health? https://www.asthmaandlung.org.uk/living-with/mental-health/looking-after (2023). Accessed 24 March 2023.

[25] Salsman ML, Nordberg HO, Christina Howell J, Berthet-Miron MM, Rosenfield D, Ritz T. Psychological distress and symptom-related burnout in asthma during the COVID-19 pandemic. J Behav Med. 2023;46(6):960–72. 37227673 10.1007/s10865-023-00412-y PMC10211287

[26] Kwong ASF, Pearson RM, Adams MJ, Northstone K, Tilling K, Smith D, et al. Mental health before and during the COVID-19 pandemic in two longitudinal UK population cohorts. Br J Psychiatry. 2021;218(6):334–43. 10.1192/bjp.2020.242 33228822 10.1192/bjp.2020.242 PMC7844173

[27] Lehrer PM, Karavidas MK, Lu SE, Feldman J, Kranitz L, Abraham S, Sanderson W, Reynolds R. Psychological treatment of comorbid asthma and panic disorder: a pilot study. J Anxiety Disord. 2008;22(4):671–83. 10.1016/j.janxdis.2007.07.001 17693054 10.1016/j.janxdis.2007.07.001 PMC2517172

[28] McDonald VM, Vertigan AE, Gibson PG. How to set up a severe asthma service. Respirology. 2011;16(6):900–11. 10.1111/j.1440-1843.2011.02012.x 21692918 10.1111/j.1440-1843.2011.02012.x

[29] European, Commission. Directorate-General for Employment, Social Affairs and Inclusion, Vanhercke B, Bouget D, Spasova S. Sick pay and sickness benefit schemes in the European Union: background report for the Social Protection Committee’s: in-depth review on sickness benefits, Brussels, 17 October 2016. Publications Office; 2016. Available from: 10.2767/531076

[30] TUC. TUC, Sick pay that works. https://www.tuc.org.uk/research-analysis/reports/sick-pay-workshttps://www.tuc.org.uk/research-analysis/reports/sick-pay-works. (2021). Accessed 15 March 2023.

[31] Chen H, Blanc PD, Hayden ML, Bleecker ER, Chawla A, Lee JH. Assessing Productivity loss and activity impairment in severe or difficult-to-treat asthma. Value Health. 2008;11(2):231–9. 18380635 10.1111/j.1524-4733.2007.00229.x

[32] Foster JM, McDonald VM, Guo M, Reddel Helen K. I have lost in every facet of my life: the hidden burden of severe asthma. Eur Respir J. 2017;50(3):1700765. 28931662 10.1183/13993003.00765-2017

[33] McClellan VE, Garrett JE. Asthma and the employment experience. PubMed. 1990;103(896):399–401. 2143568

[34] Ilmarinen J. Work ability—a comprehensive concept for occupational health research and prevention. Scand J Work Environ Health. 2009;35(1):1–5. 19277432 10.5271/sjweh.1304

[35] Walters GI, McGrath EE, Ayres JG. Audit of the recording of occupational asthma in primary care. Occup Med. 2012;62(7):570–3. 10.1093/occmed/kqs114 22837332

[36] Barber CM, Cullinan P, Feary J, Fishwick D, Hoyle J, Mainman H, et al. British thoracic Society Clinical Statement on occupational asthma. Thorax. 2022;77(5):433–42. 35314486 10.1136/thoraxjnl-2021-218597

[37] Henneberger PK, Redlich CA, Callahan DB, Harber P, Lemière C, Martin J, et al. An official American thoracic Society Statement: Work-Exacerbated Asthma. Am J Respir Crit Care Med. 2011;184(3):368–78. 21804122 10.1164/rccm.812011ST

[38] Walters G, Reilly C, Le Moual N, et al. Asthma control in severe asthma and occupational exposures to inhaled asthmagens. Eur Respir J. 2023;62(Suppl67):PA3348. 10.1183/13993003.congress-2023.PA3348

[39] Jackson DJ, Busby J, Pfeffer PE, Menzies-Gow A, Brown T, Gore R, et al. Characterisation of patients with severe asthma in the UK severe Asthma Registry in the biologic era. Thorax. 2020;76(3):220–7. 33298582 10.1136/thoraxjnl-2020-215168 PMC7892381

[40] Busby J, Heaney LG, Brown T, Chaudhuri R, Dennison P, Gore R et al. Ethnic Differences in Severe Asthma Clinical Care and Outcomes: An Analysis of United Kingdom Primary and Specialist Care. The Journal of Allergy and Clinical Immunology: In Practice. 2022;10(2):495–505.e2. Available from: https://www.sciencedirect.com/science/article/abs/pii/S2213219821010618?dgcid=rss_sd_all 10.1016/j.jaip.2021.09.034 34626858

[41] Taylor PL, Tucker MB, Mitchell-Kernan C. Ethnic variations in perceptions of men’s provider role. Psychol Women Q. 1999;23(4):741–61.

[42] GOV.UK. Benefits and financial support if you’re disabled or have a health condition. https://www.gov.uk/browse/benefits/disability. Accessed on 13 April 2023.

[43] Asthma UK. Managing your lung condition at work. https://www.asthmaandlung.org.uk/living-with/working-lung-condition. (2023). Accessed on 15 April 2023.

[44] Shenolikar R, Song X, Anderson JA, Chu BC, Cantrell CR. Costs of asthma among US working adults. Am J Manag Care. 2011;17(6):409–16. 21756011

