# Supplementary material for: ‘Stuck in catch-22’: a qualitative study of perceived work ability and decision-making about employment in severe asthma
Source: BMC Pulm Med. 2025 Feb 17;25:82. doi: 10.1186/s12890-025-03499-y (PMC11834327; doi:10.1186/s12890-025-03499-y)
Supplement: Supplementary file 1 — Supplementary Material 1 [file 12890_2025_3499_MOESM1_ESM.docx]

**Supplementary file.**

**Interview schedule developed for this study**

Domain 1: Demographics

1. How old are you?

2. How would describe your ethnicity?

3. Do you smoke cigarettes, and/or other, and/or drink alcohol, and how often?

1. Has this affected your ability to work? (If so, could you tell me how?

4. Do you have any allergies? If so, how do they affect you? (drugs, aeroallergens, foodstuffs)

5. Can you briefly describe your education to date, and any further training or education since you left school?

6. How long have you been working for your current employer?

Or how long have you been working for your past employer? How long have you been out of work?

1. Describe your job role
2. Terms (paid or unpaid, full-time or part-time/casual)
3. Patterns of work (shifts, lone working, unsociable hours)
4. Has covid-19 impacted on your work? If so, how? (Patterns, hours, working from home)?

7. Do you have any dependents or caring responsibilities?

1. Can you describe your responsibilities?
2. Do you share these with anyone?
3. Who is at home with you?
4. Does your asthma treatment impact on your caring responsibilities? If yes, tell me a bit more how?
5. In turn, does this impact on your work? If yes, tell me a bit more how?

Domain 2: Asthma-specific beliefs and experiences

1. Do your asthma symptoms impact on your ability to work?

1. Please be specific on how you are affected and when (all the time, periodically, specific times)
2. Can you tell me whether there are any tasks that you are unable to do effectively at work?
3. If yes, how do you manage these disabilities?
4. How does this make you feel about going to, and being at, work?

2. Have you taken time off work because of asthma symptoms?

1. Elaborate on frequency, cause (exacerbations, day to day symptoms)
2. Any hospitalisations or emergency room visits?
3. Elaborate on return-to-work process

3. Have your asthma treatments affected your ability to go to work and do your job? If so, how? (specific about appointments, drug delivery, hospital or primary care treatments, side effects)

Domain 3: General physical and psychological health status

1. What else do you think causes your inability to work/unemployment? (Any social, cultural factors?) Can you elaborate how these factors that you mentioned affect it?

2. Do you have any other (not asthma) medical problems? Do they limit your ability to do your job?

- 1. If yes, how do they affect you, and which tasks are inhibited?
  2. Elaborate on constitutional symptoms eg. fatigue, myalgia, insomnia
  3. Specifically: musculoskeletal, obesity, reflux, OSA
  4. Specifically: low mood or feeling anxious

3. How do you rate your quality of life? Why have you rated it as good/bad?

1. Does that affect your ability to work? If so, how? (e.g., mental, physical, or environmental berries?
2. In turn do you feel like your work ability/disability affects your quality of life?
3. Has your quality of life impacted on your ability to enjoy work?

Domain 4: Workplace exposures, beliefs about the nature of work

1. Are there any exposures at work that make your asthma worse? (physical triggers, including cold air and exercise, airway irritants, emotional stress)

2. Does your employer understand your conditions (severe asthma and associated diagnoses)?

1. What is your relationship with human resources/your line manager, and with occupational health provider (if exists)?

3. Are you able to have a say in how you manage your workload and timetable?

4. Do you feel valued at work? (if not, how does your asthma impact on this?)

5. Is the content of your job meaningful? (any change because of your asthma?)

6. Do you enjoy your work? (how does your asthma impact on enjoyment?)

7. What would make doing your job easier? (adaptations eg. timetabling, process adjustments)

8. Why do you go to work? (elaborate, including: financial reward, growth, promotion)

9. Do you believe that work is good for you?

10. Would it be easy to find another job if you needed to?

If out of work but have worked in the past - same questions in the past tense.

**Supplementary Table.**

**Codes from which the final high-level themes and subthemes were constructed**

| Theme | Sub-theme | Code |
| --- | --- | --- |
| Impact of patients' asthma control on work | Asthma symptoms and productivity | Work struggles with poorly managed asthma |
|  |  | Absenteeism – taking time off to recover |
|  |  | Presenteeism – working while unwell |
|  |  | Return to work after sickness |
|  |  | Increased work capacity with good asthma management |
|  | Asthma triggers | Seasonal asthma worsening |
|  |  | Environmental exposures |
|  |  | Work stress |
|  | Impact of asthma treatment and its perceived effectiveness | Finding suitable treatment |
|  |  | Side effects of medication |
|  |  | Caution and routine |
|  |  | Brittle clinical features |
|  |  | Medication administration at work |
|  |  | Biologic injections |
| Psychological burden of living with severe asthma | Effects of traumatic asthma attacks on work | Anticipation of an asthma attack – intrusive thoughts, anxiety, and panic |
|  |  | Embarrassment, avoidance and isolation |
|  | Chronic illness burnout | Frustration about asthma limitations and affected social roles |
|  |  | Low self-worth and confidence |
|  |  | Tiredness and motivation |
|  |  | Mental health stigma |
| Cost and benefits of being in employment | Coping financially | Pressure to pay the bills |
|  |  | Sick pay and benefits entitlement |
|  |  | Reduced hours |
|  |  | Attitude towards benefits |
|  |  | Societal pressure to work |
|  | Work relationships | Managers awareness of impact of severe asthma on work |
|  |  | Colleagues understanding of severe asthma (distinct condition) |
|  |  | Return to work and absence warnings - feeling threatened about job stability |
|  |  | Discrimination in the workplace |
|  | Job satisfaction | Work ethics |
|  |  | Meaning and purpose in life |
|  |  | Fear of identity and independence loss |
|  |  | Feeling valued |
| Adaptations and strategies for remaining in employment | Managing work expectations - patient initiated adaptations | Recognising and accepting asthma limitations - health comes first |
|  |  | Carrying on as normal – shame, guilt and anger |
|  |  | Confidence building |
|  | Structural changes – employer-initiated adjustments | Reasonable adjustments under the Equality Act 2010 |
|  |  | Changing hours |
|  |  | Changing routine |
|  |  | Accessibility |
|  |  | Changing role or relocation |
|  |  | Flexible working |
|  |  | Self-employment |
|  | Support from family and friends | Financial help when off sick |
|  |  | Practical help with work tasks |
|  |  | Emotional help to deal with asthma challenges |
